# Serum 25-Hydroxyvitamin D3 and BAFF Levels Are Associated with Disease Activity in Primary Sjogren's Syndrome

**DOI:** 10.1155/2016/5781070

**Published:** 2016-12-15

**Authors:** Sang Jin Lee, Hye Jin Oh, Byoong Yong Choi, Yu Jin Jang, Joo Youn Lee, Jin Kyun Park, Yeong Wook Song

**Affiliations:** ^1^Division of Rheumatology, Department of Internal Medicine, Seoul National University Hospital, Seoul, Republic of Korea; ^2^Department of Molecular Medicine and Biopharmaceutical Sciences, Graduate School of Convergence Science and Technology, and College of Medicine, Medical Research Center, Seoul National University, Seoul, Republic of Korea; ^3^Division of Rheumatology, Department of Internal Medicine, Seoul Medical Center, Seoul, Republic of Korea

## Abstract

The study investigated the association between disease activity and serum 25-hydroxyvitamin D3 (25(OH)-D3), B cell activation of the tumor necrosis factor family (BAFF), or *β*_2_ microglobulin in patients with primary Sjogren's syndrome (SS). Sixty-nine primary SS patients and 22 sicca control patients were included in the study. Disease activity was measured with EULAR Sjogren's syndrome disease activity index (ESSDAI). Serum levels of 25(OH)-D3 and *β*_2_ microglobulin were measured by radioimmunoassay and BAFF was measured by an enzyme-linked immunosorbent assay. Serum levels of 25(OH)-D3 were significantly lower in SS patients compared to the sicca controls (*p* = 0.036). Serum levels of BAFF tended to be higher (*p* = 0.225) and those of *β*_2_ microglobulin were significantly higher in patients with SS than in sicca controls (*p* = 0.023). In univariate regression analyses, ESSDAI was significantly associated with serum levels of 25(OH)-D3, BAFF, and *β*_2_ microglobulin. After stepwise backward multivariate linear regression analyses including age and acute phase reactants, ESSDAI was associated with 25(OH)-D3 (*β* = −0.042, *p* = 0.015) and BAFF (*β* = 0.001, *p* = 0.015) in SS patients. In SS patients, ESSDAI is negatively associated with serum levels of 25(OH)-D3 and positively associated with BAFF.

## 1. Introduction

Sjogren's syndrome (SS) is a chronic autoimmune disease affecting the exocrine glands that manifests as sicca symptoms including dry eyes and dry mouth. SS often involves extraglandular organs including joints, liver, lung, brain, and kidney. The extraglandular manifestations are mediated in part by the overproduction of multiple autoantibodies that are often directed against nuclear antigens such as antinuclear antibody (ANA) and anti-Ro/La antibodies, which lead to hypergammaglobulinemia due to chronic polyclonal B cell activation [1]. Lymphocytes or autoantibodies lead to the inflammation of the target tissues directly or due to the formation of immune complexes. Chronic B cell activation plays an important role in the pathogenesis of SS [2]. Factors associated with B cell activation were reported to correlate with SS disease activity; these include serum levels of B cell activating factor belonging to the tumor necrosis factor family (BAFF) [3], *β*_2_ microglobulin [4, 5], and free light chains of immunoglobulin [6].

Vitamin D3 has an immunomodulatory function [7]. Low serum vitamin D3 levels have been associated with several autoimmune diseases including multiple sclerosis, type 1 diabetes mellitus, rheumatoid arthritis (RA), systemic lupus erythematous (SLE), Behcet's disease (BD), and idiopathic inflammatory myopathies [8–13]. In RA and SLE patients, vitamin D3 levels negatively correlate with disease activity [14, 15]. Data on vitamin D3 levels in SS have been conflicting. Some studies reported that vitamin D3 levels were similar between SS patients and healthy controls [16] and did not correlate with EULAR Sjogren's syndrome disease activity index (ESSDAI), but the number of enrolled SS patients was small (*n* = 30) [17]. Other studies showed that vitamin D3 levels were significantly lower in SS patients compared to healthy controls [18] and low levels of vitamin D3 were associated with the presence of peripheral neuropathy and lymphoma [19].

The aim of the present study was to investigate the association between SS disease activity and serum 25(OH)-D3, BAFF, and *β*_2_ microglobulin.

## 2. Material and Methods

### 2.1. Study Population

Sixty-nine patients with primary SS according to the American-European Consensus Classification Criteria [20] and 22 age- and sex-matched patients with sicca as a control group were recruited from the Rheumatology Clinic of Seoul National University Hospital. Primary SS patients did not have any other autoimmune diseases and the control patients had dry mouth and/or dry eyes but were not diagnosed with SS. This study was performed between November 2012 and February 2013. The study protocol was approved by the Ethics Committee of Seoul National University Hospital. All patients gave informed consent.

### 2.2. Assessment of SS Activity and Serological Parameters

Clinical and laboratory data were obtained from medical records. Disease activity of SS expressed as ESSDAI was ascertained at the time of blood sampling [21]. Serum levels of 25(OH)-D3 and *β*_2_ microglobulin were measured by radioimmunoassay (RIA) (Immunotech, Sao Paulo, Brazil, and DiaSorin, Minneapolis, MN, USA, resp.). Serum levels of BAFF were determined by enzyme-linked immunosorbent assay (ELISA) (R&D Systems, Minneapolis, MN, USA). Serum antinuclear antibody (ANA) was detected by indirect immunofluorescence (Bio-Rad, Hercules, CA, USA), anti-Ro/La antibodies were detected by ELISA (Zeus Scientific, Somerville, NJ, USA), and RF was detected by immunoturbidimetry (Roche, Mannheim, Germany).

### 2.3. Statistical Analyses

Data are expressed as mean ± standard error of mean (SEM) for continuous variables and as percentages for categorical variables. The Student* t*-test was used to compare continuous variables and chi-square test or Fisher's exact test was used to compare categorical variables. Correlation between ESSDAI scores and levels of serological parameters was analyzed by Spearman's correlation. Multivariate analyses by the stepwise backward method were used to evaluate the association between serological parameters and ESSDAI. *p* values < 0.05 were considered statistically significant. All statistical analyses were performed using SPSS version 19 software (IBM, Chicago, IL, USA) and graphics were generated in GraphPad Prism version 5 (GraphPad, San Diego, CA, USA).

## 3. Results

### 3.1. Clinical and Laboratory Characteristics of Patients with SS and Sicca

Sixty-nine primary SS patients and 22 sicca patients were enrolled. The mean age (±SEM) of the SS and sicca patients was 56.7 ± 1.32 and 58.0 ± 2.66 years, respectively. The majority of both groups were females (98.6% in the SS group, 95.5% in the sicca group). The mean duration after diagnosis was 8.7 ± 0.78 and 5.7 ± 1.09 years, respectively. Serum autoantibody positive rates were significantly higher in SS patients than in sicca patients: ANA 87.0% versus 13.6%, anti-Ro (SSA) 91.3% versus 0.0%, and anti-La (SSB) 62.3% versus 0.0% (all, *p* < 0.001). The mean ESSDAI was 1.5 ± 0.17 in SS patients and none in sicca patients (Table 1). In SS, all patients took hydroxychloroquine. In addition, 36.2% (*n* = 25) and 17.4% (*n* = 12) of patients received nonsteroidal anti-inflammatory drugs (NSAIDs) and low dose steroids (prednisolone equivalent ≤ 10 mg/day), respectively.

### 3.2. Erythrocyte Sedimentation Rate (ESR) and Levels of Serum C-Reactive Protein (CRP), 25(OH)-D3, BAFF, and *β*_2_ Microglobulin in SS and Sicca Patients

ESR (26.6 ± 2.50 versus 11.5 ± 1.64 mm/hr, respectively; *p* < 0.001) and *β*_2_ microglobulin (1.57 ± 0.08 versus 1.19 ± 0.08 mg/L, respectively; *p* = 0.023) were significantly higher in SS patients compared to sicca patients. CRP levels were not significantly different in both groups (0.24 ± 0.06 versus 0.13 ± 0.10 mg/dL, respectively; *p* = 0.368). 25(OH)-D3 levels were significantly decreased in SS patients compared to sicca patients (22.0 ± 1.32 versus 28.0 ± 2.69 ng/mL, respectively; *p* = 0.036). Levels of BAFF tended to be higher, albeit nonsignificant, in SS patients compared to sicca patients (1543 ± 141 versus 1200 ± 182 pg/mL, respectively; *p* = 0.225) (Table 1). There were no significant differences in levels of 25(OH)-D3, BAFF, and *β*_2_ microglobulin according to medications (NSAIDs or steroids in patients with SS, data not shown).

Extraglandular involvement was present in 35 patients of SS (50.7%). We analyzed age and serological parameters according to extraglandular organ involvement in SS patients. ESSDAI and levels of CRP and BAFF were significantly higher in the group of extraglandular involvement. But levels of 25(OH)-D3 tended to be lower in this group (see Supplementary Table  1 in Supplementary Material available online at http://dx.doi.org/10.1155/2016/5781070).

### 3.3. Correlation between ESSDAI Scores and Serological Parameters

**Figure 1:**
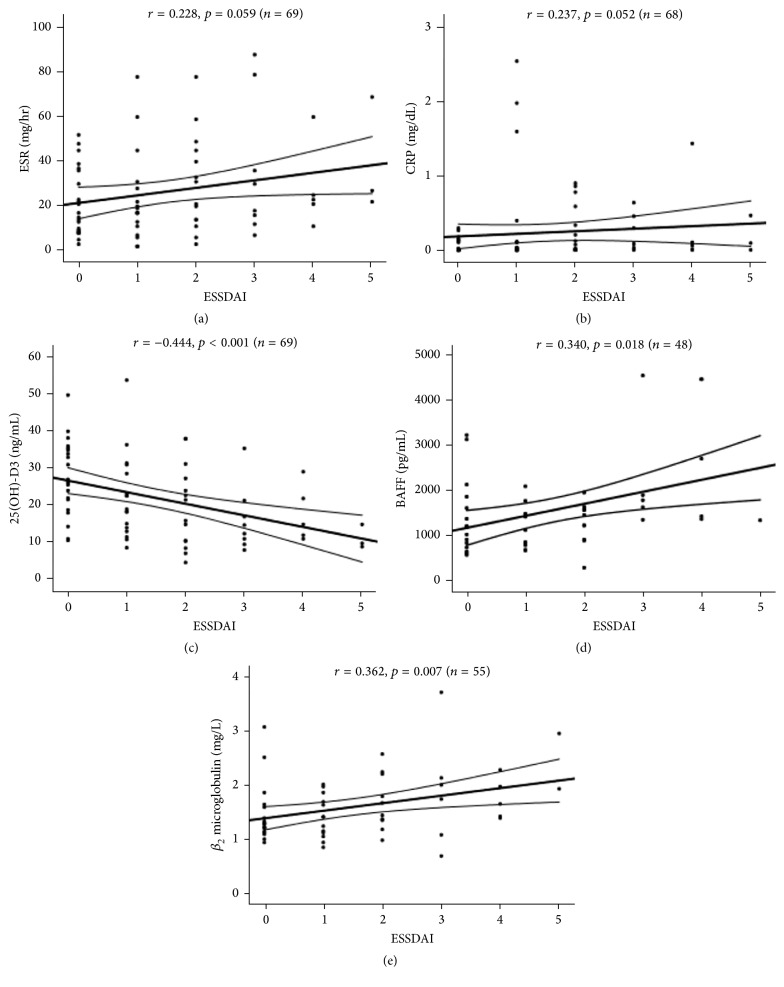
Correlation of ESSDAI with levels of ESR, serum CRP, 25(OH)-D3, BAFF, and *β*_2_ microglobulin in patients with primary Sjogren's syndrome. Each dot represents individual value (correlation coefficient and *p* value by Spearman's rank correlation test).

ESSDAI tended to correlate with levels of ESR (*r* = 0.228, *p* = 0.059) or CRP (*r* = 0.237, *p* = 0.052). ESSDAI was inversely correlated with serum levels of 25(OH)-D3 (*r* = −0.444, *p* < 0.001) (Figure 1). But levels of 25(OH)-D3 were not significantly correlated with age, ESR, levels of CRP, BAFF, and *β*_2_ microglobulin in SS patients (Table 2). There was a positive correlation between ESSDAI and levels of BAFF (*r* = 0.340, *p* = 0.018) or *β*_2_ microglobulin (*r* = 0.362, *p* = 0.007) (Figure 1). Levels of BAFF were not significantly correlated with age, levels of CRP, 25(OH)-D3, and *β*_2_ microglobulin, except ESR in SS patients. Levels of *β*_2_ microglobulin were not significantly correlated with age, levels of 25(OH)-D3, and BAFF, except ESR and levels of CRP in SS patients (Table 2).

### 3.4. Associations of ESSDAI with Serological Parameters by Univariate and Multivariate Linear Regression

In univariate regression analyses, 25(OH)-D3, BAFF, and *β*_2_ microglobulin were significantly associated with ESSDAI. To identify factors associated with ESSDAI, multivariate linear regression analyses were performed considering age, ESR, and CRP in addition to 25(OH)-D3, BAFF, and *β*_2_ microglobulin. ESSDAI was associated with 25(OH)-D3 (*β* = −0.042, *p* = 0.015) and BAFF (*β* = 0.001, *p* = 0.015), but not with age (*β* = −0.036, *p* = 0.076) (Table 3).

## 4. Discussion

In the present study, serum levels of 25(OH)-D3 were significantly lower and those of *β*_2_ microglobulin were significantly higher in patients with SS compared with age- and sex-matched sicca controls. Levels of BAFF tended to be higher in SS patients. Consistent with previous reports [3, 5], our data showed that ESSDAI was correlated with serum beta2-microglobulin in SS patients in univariate analyses. But in multivariate analyses including serum levels of 25(OH)-D3, BAFF, and *β*_2_ microglobulin, ESSDAI was associated with 25(OH)-D3 and BAFF but not with *β*_2_ microglobulin. These suggest that both serum levels of 25(OH)-D3 and BAFF are independent predictors of ESSDAI in SS patients.

There is limitation in this study. Disease activity of enrolled patients was relatively low. Larger studies of SS patients with higher disease activity may be needed for further validation.

BAFF enhances B cell maturation, proliferation, and survival [1]. Serum levels of BAFF are increased in patients with autoimmune diseases such as SLE, SS, and rheumatoid arthritis [22]. It was reported that elevated serum BAFF levels were independent predictor of flare in patients who were receiving standard SLE therapy [23]. BAFF upregulation was associated with B cell clonal expansion in the salivary gland and correlated with SS disease activity [3, 4]. Long-term treatment with belimumab, a fully human monoclonal antibody direct against BAFF, decreased disease activity and was safe in both SLE and SS patients [24, 25]. *β*_2_ microglobulin forms the light chain of MHC class I molecules that are crucial for antigen presentation to T cell receptor. Serum levels of *β*_2_ microglobulin were associated with extraglandular involvement such as renal or pulmonary manifestations in patients with SS [26, 27].

Low levels of 25(OH)-D3 have been associated with lymphoma and observed in SS patients over 66 years of age compared to patients below 50 years of age [19]. There were no patients with lymphoma enrolled in our study and SS patients >66 years of age tended to have lower levels of 25(OH)-D3 than those <50 years of age (19.27 ± 2.44 versus 24.06 ± 2.81; *p* = 0.285). However, when we used multivariate linear regression analyses including age, ESSDAI was associated with 25(OH)-D3 but not with age.

Vitamin D may play an immunomodulatory role in both innate and adaptive immunity [28]. 1,25(OH)2D3 suppresses Toll-like receptor- (TLR-) 2 and TLR-4 expression in human monocytes, leading to hyporesponsiveness to pathogen-associated molecular patterns [29, 30]. Specifically, this hormone inhibits TLR-2 and TLR-4 expression of monocytes in BD patients in a dose-dependent manner [31]. 1,25(OH)2D3 promotes a shift in the T helper (Th)1/Th2 balance toward Th2 and downregulates Th17 autoimmunity by reducing interleukin-17A-secreting CD4+ T cells [32, 33]. A highly significant negative correlation between levels of 25(OH)-D3 and IgG levels in healthy female was reported [34]. In addition 1,25(OH)2D3 level was associated with reduced human B cell proliferation, plasma cell differentiation, and IgG production from peripheral blood in vitro [35]. Activation of B cells is crucial and the risk of lymphoma is increased in SS compared to the general population [1, 2]. B cell targeted agents, such as rituximab and belimumab, significantly improved ESSDAI compared to placebo treatment in SS patients [36]. Our results suggest that low levels of 25(OH)-D3 are associated with increased systemic disease activity in SS patients.

Low vitamin D levels were associated with many rheumatic diseases such as RA, SLE, BD, and idiopathic inflammatory myopathies [10–13]. Recently, systematic review and meta-analysis have highlighted associations between vitamin D deficiency and ankylosing spondylitis and inflammatory bowel disease [37, 38].

Long-term administration of vitamin D3 analogs in mice decreases the levels of serum IgG and interleukin-2, which are crucial in the immune system [39]. Vitamin D deficiency is common in patients with rheumatic disease particularly SLE [40]. After vitamin D supplementation modest improvement of disease activity [41, 42] and restoration of B cell homeostasis were observed in SLE [43]. However other studies showed that vitamin D supplementation failed to affect disease activity and diminish the interferon signature in patients with SLE [44, 45]. Additionally, replacement of vitamin D improved endothelial function in BD patients [46]. Supplementation of vitamin D may be considered in patients with SS and lower vitamin D levels.

## 5. Conclusions

Serum levels of 25(OH)-D3 were significantly lower in patients with SS compared with age- and sex-matched sicca controls. ESSDAI is negatively associated with serum levels of 25(OH)-D3 and positively associated with BAFF in patients with SS.

## Supplementary Material

Age and serological parameters according to extra-glandular organ involvement in patients with primary Sjogren's syndrome.

## Figures and Tables

**Table 1:** Clinical and laboratory characteristics of primary Sjogren's syndrome and sicca patients.

Features	Sjogren's syndrome (*n* = 69)	Sicca (*n* = 22)	*p* value
Age at diagnosis, years	56.7 ± 1.32	58.0 ± 2.66	0.651
Sex, female (%)	68 (98.6)	21 (95.5)	0.427
BMI (kg/m^2^)	21.9 ± 0.34	22.5 ± 0.63	0.356
Disease duration, years	8.7 ± 0.78	5.7 ± 1.09	0.047^*∗*^
Anti-Ro (SSA), *n* (%)	63 (91.3)	0 (0.0)	<0.001^*∗*^
Anti-La (SSB), *n* (%)	43 (62.3)	0 (0.0)	<0.001^*∗*^
Antinuclear antibody, *n* (%)	60 (87.0)	3 (13.6)	<0.001^*∗*^
Rheumatoid factor, *n* (%)	37/64 (57.8)	7 (31.8)	0.043^*∗*^
ESR (mm/hr)	26.6 ± 2.50	11.5 ± 1.64	<0.001^*∗*^
CRP (mg/dL)	0.24 ± 0.06	0.13 ± 0.10	0.365
25(OH)-D3 (ng/mL)	22.0 ± 1.32	28.0 ± 2.69	0.036^*∗*^
BAFF (pg/mL)	1543 ± 141	1200 ± 182	0.225
*β*_2_ microglobulin (mg/L)	1.57 ± 0.08	1.19 ± 0.08	0.023^*∗*^
Extraglandular involvement (%)	35 (50.7)	0	
ESSDAI	1.5 ± 0.17	0	

Data are presented as mean ± SEM for continuous data and number (percentage) for categorical variables. BMI = body mass index; ESR = erythrocyte sedimentation rate; CRP = C-reactive protein; BAFF = B cell activation of the TNF family; ESSDAI = EULAR Sjogren's syndrome disease activity index; ^*∗*^ *p* < 0.05.

**Table 2:** Correlations between serum levels of 25(OH)-D3, BAFF, or *β*_2_ microglobulin and serological parameters were evaluated in primary Sjogren's syndrome.

	25(OH)-D3		BAFF		*β*_2_ microglobulin	
	Correlation efficient	*p* value	Correlation efficient	*p* value	Correlation efficient	*p* value
Age	−0.110	0.366	0.101	0.493	0.233	0.086
ESR	0.174	0.153	0.311	0.031^*∗*^	0.498	<0.001^*∗*^
CRP	−0.009	0.941	0.281	0.055	0.296	0.030^*∗*^
25(OH)-D3			−0.028	0.849	−0.096	0.485
BAFF	−0.028	0.849			0.215	0.147
*β*_2_ microglobulin	−0.096	0.485	0.215	0.147		

Correlation coefficient and *p* value were analyzed by Spearman's rank correlation test. ESR = erythrocyte sedimentation rate; CRP = C-reactive protein; BAFF = B cell activation of the TNF family; ^*∗*^ *p* < 0.05.

**Table 3:** Associations of ESSDAI with serological parameters by univariate and multivariate linear regression.

Variate	Univariate analyses		Multivariate analyses (backward stepwise)	
	*β* ± SE	*p* value	*β* ± SE	*p* value
Age (years)	−0.006 ± 0.016	0.732	−0.036 ± 0.020	0.076
ESR (mm/hr)	0.017 ± 0.008	0.050		
CRP (mg/dL)	0.320 ± 0.374	0.396		
25(OH)-D3 (ng/mL)	−0.055 ± 0.015	<0.001^*∗*^	−0.042 ± 0.016	0.015^*∗*^
BAFF (pg/mL)	0.001 ± 0.000	0.007^*∗*^	0.001 ± 0.000	0.015^*∗*^
*β*_2_ microglobulin (mg/L)	0.865 ± 0.321	0.009^*∗*^		

ESSDAI = EULAR Sjogren's syndrome disease activity index; ESR = erythrocyte sedimentation rate; CRP = C-reactive protein; BAFF = B cell activation of the TNF family; ^*∗*^ *p* < 0.05.
