# Supplementary material for: Serum 25-Hydroxyvitamin D3 and BAFF Levels Are Associated with Disease Activity in Primary Sjogren's Syndrome
Source: J Immunol Res. 2016 Dec 15;2016:5781070. doi: 10.1155/2016/5781070 (PMC5198174; doi:10.1155/2016/5781070)
Supplement: Supplementary file 1 — Age and serological parameters according to extra-glandular organ involvement in patients with primary Sjogren's syndrome. [file 5781070.f1.pdf]

Supplementary table 1. Age and serological parameters according to extra-glandular organ involvement in patients with primary Sjogren's syndrome

	Extra-glandular involvement (+) (n=35)	Extra-glandular involvement (-) (n=34)	p-value
Age (years)	57.0 ± 1.77	56.4 ± 1.99	0.904
ESR (mm/hr)	31.0 ± 3.97	22.0 ± 2.87	0.068
CRP (mg/dl)	0.38 ± 0.11	0.11 ± 0.03	0.024*
25(OH)-D3 (ng/ml)	20.9 ± 1.84	23.2 ± 1.89	0.325
BAFF (pg/ml)	1823 ± 238	1285 ± 144	0.028*
β_2 microglobulin (mg/l)	1.70 ± 0.13	1.45 ± 0.09	0.102
ESSDAI	2.4 ± 0.23	0.6 ± 0.13	<0.001*

Data are presented as mean ± SEM for continuous data. ESR = erythrocyte sedimentation rate; CRP = C-reactive protein; BAFF = B-cell activation of the TNF family; ESSDAI = EULAR Sjogrens' syndrome disease activity index; *p<0.05.
